# 

**Published:** 1896-09

**Authors:** 


### CONTENTS.

ORIGINAL COMMUNICATIONS—
A FURTHER CONTRIBUTION TO THE USE OF THE GALVANO-CAUTERY IN PTERYGIUM OPERATIONS. By Arthur G. Hobbs, M.D., Atlanta, Ga. .... 433
REMARKS ON THE TECHNIQUE OF EXTERNAL URETHROTOMY. By W. S. Goldsmith, M.D., Atlanta, Ga. .... 437
A FEW POINTS IN THE TREATMENT OF MORPHINISM. By C. C. Stockard, M.D., Atlanta, Ga. .... 444
STRANGULATED HERNIA. By Virginius W. Harrison, A.M., M.D., Richmond, Va. .... 446
CÆSAREAN OPERATION UNDER DIFFICULTIES. By I. P. Allred, M.D., Genoa, Fla. .... 452
EPIDEMIC TYPHOID FEVER. By Wm. S. Gordon, M.D., Richmond, Va. .... 454
SOCIETY REPORTS—
RICHMOND ACADEMY OF MEDICINE AND SURGERY .... 460
CORRESPONDENCE—
NEW YORK LETTER .... 468
EDITORIAL—
DRINKING WATER .... 473
THE PREVENTION OF CHOLERA .... 475
SANITARY CLOTHING .... 476
OUR DECREASING BIRTH-RATE .... 479
SELECTIONS AND ABSTRACTS—
SKIN DISEASES AND SYPHILIS .... 480
TREATMENT OF HOMOPHILIA .... 483
RECENT PROGRESS IN THE TREATMENT OF FRACTURES .... 485
MEDICAL ITEMS .... 489
AMERICAN ASSOCIATION OF OBSTETRICIANS AND GYNECOLOGISTS .... 495
BOOK REVIEWS .... 501
MEDICAL ITEMS—CONTINUED .... x, xi, xii
ADVERTISERS' INDEX TO ADVERTISEMENTS .... xiv
CLASSIFIED INDEX TO ADVERTISEMENTS .... xxxv

